# Ground-penetrating radar scanning and historical interpretation of the location of the destroyed Epiphany Cathedral in Kyiv Brotherhood Monastery (Ukraine)

**DOI:** 10.12688/openreseurope.16592.2

**Published:** 2024-06-24

**Authors:** Kseniia Bondar, Sergiy Taranenko, Yaroslav Zatyliuk, Olena Popelnytska, Tetiana Osinchuk

**Affiliations:** 1 Institute of Geology, Taras Shevchenko National University of Kyiv, Kyiv, 03022, Ukraine; 2 National Reserve «Kyiv-Pechersk Lavra», Kyiv, 01015, Ukraine; 3 Municipal Institution "Center for Conservation of Archaeological Artifacts", Department of Cultural Heritage Protection of Kyiv City State Administration, Kyiv, 04070, Ukraine; 4 Department of Archaeology and Anthropology, Bournemouth University, Poole, England, UK; 5 The National Museum of the History of Ukraine, Kyiv, 01001, Ukraine; 6 Institute of History of Ukraine National Academy of Science of Ukraine, Kyiv, 01001, Ukraine

**Keywords:** ground penetrating radar, hidden foundation, Kyiv Brotherhood monastery, Kyiv-Mohyla Academy, Epiphany Cathedral, Hetman Sahaidachny`s burial site

## Abstract

**Background:**

The article presents results of a ground penetrating radar (GPR) scanning carried out in a site of the Epiphany Cathedral of Brotherhood Monastery in Kyiv, Ukraine, destroyed in 1936 by the Soviets. The Cathedral is known as a burial site of Hetman Petro Sahaidachnyi (1582–1622), a Ukrainian Cossack political and civic leader, guardian and patron of Kyiv Brotherhood Monastery. The collection of archival documents, blueprints, stock sources, photographs and cartographic materials of the 17th-19th centuries, as well as historical works of the 19th-21st centuries, were thoroughly analyzed and used as a basis for the interpretation of geophysical results. The set of historical data covers the period from 1615, that is, from the beginning of the construction of the wooden Epiphany Church, the predecessor of the cathedral, to the present day.

**Methods:**

Summarized information about the construction, restoration after the fire, functioning and destruction of the Cathedral, as well as about the construction on its site in the 20th century, archaeological research in the 20th-21st centuries, was used to clarify possible location of hidden foundations and target GPR measurements. In this context, written testimonies of archaeologists who personally observed the foundations of the cathedral became especially valuable. The shielded GPR antennas, with a central frequency of 300 MHz and 700 MHz, is used for non-invasive investigation.

**Results:**

GPR scanning specified the site and showed the best-preserved group of foundations of the western wall of the Epiphany Cathedral.

**Conclusions:**

An integrated historical and geophysical study provided the basis to certify the foundation of the Epiphany Cathedral as a protected object of cultural heritage and facilitated its archaeological research.

## Plain language summary

A comprehensive understanding of the significance and archaeological potential of the site of destroyed Epiphany Cathedral of Kyiv Brotherhood Monastery was established by combining historical research with ground penetrating radar technique. The historical research provides context and background information about the Cathedral, including its construction, renovations, previous uses, and any known archaeological discoveries. Two locations of Cathedral's foundations were identified as well as excavated area nearby, which did not reveal remains of the structure. The analytical approach implemented in this paper allowed us to precisely target GPR measurements. In this study, a dual-frequency GPR system (300 MHz and 700 MHz) was used. The 300 MHz antenna proved to be effective in penetrating clay-rich cultural layers studied at a depth of 2.0 m. This allowed us to image and separate the city communications and fragments of the foundation in the studied area in the radargrams and C-scans. The ground-penetrating radar survey showed the best preservation of the foundations of the massive western wall of the Cathedral. The proposed reconstruction of the location of Cathedral`s foundation is to be used in the development of archaeological investigation, conservation and management plans for the site, ensuring its protection and preservation.

## Introduction

In 1936, the architectural complex of Kyiv Brotherhood Monastery lost its main shrine - the Epiphany Cathedral. It was dismantled as having no cultural and artistic value during the socialist reconstruction of Kyiv ( Gevryk, 1990; Khaustov, 1936; Kilesso, 2002).

However, since 1991, which marked the end of the Soviet era, a possibility of restoring this architectural pearl has been considered at the government level with variable success. A significant difficulty in solving the issue of reproducing the Cathedral is the lack of an exhaustive and sufficient information on its position, interior and underground structure which could be obtained from thorough archaeological research.

The task of precise localization of a hidden architectural structure, of which there are no visible traces, requires a complex approach. A special place in such research is given to non-destructive geophysical methods, which allow us to find the remains of buried foundations, if they are at least partially preserved ( Forte *et al*., 2021; Deiana *et al*., 2018; Lück *et al*., 1997). The choice of the appropriate geophysical technology is dictated by natural and man-made factors and local conditions - the type of soil, the nature of vegetation, proximity to buildings and power lines, the presence of asphalt pavement and electromagnetic interference. As world experience shows, in the conditions of urban development, the most effective way to find underground heterogeneities is ground-penetrating radar (GPR) scanning ( Dwivedi *et al*., 2022; Leucci & Negri, 2006; Ristić *et al*., 2020). The use of modern shielded antennas to determine underground targets is possible both in the open air and inside buildings. The presence of nearby industrial enterprises and busy highways also, for the most part, do not create significant obstacles. At the same time, the structure, type and degree of soil moisture impose additional restrictions, in particular, on the depth of the method ( Conyers, 2017; Goodman & Piro, 2013).

A necessary condition for the successful application of geophysical technologies is the justified choice of the research site, which is made on the basis of a careful analysis of cartographic materials and an assessment of data from historical documents. It is important to collect as much reliable spatial information as possible with the most accurate reference to the area. However, one should not neglect even unreliable data that can contribute the research and do not require significant effort for instrumental verification in the field. Therefore, the collection and analysis of archival and published information, as well as cartographic materials, became an integral part of this study.

There is an extensive literature discussing the use of geophysical techniques to reveal architectural structures hidden in the heterogeneous subsurface of urban environments. Ristić *et al.* (2020) reported a very good accuracy for the identification of ancient gate remnants later excavated based on the GPR findings. The authors insist GPR should be incorporated as a routine field procedure in construction and renovation projects involving historical cities. Bondar and Vietrov (2023) claim the GPR survey was capable of seeing elements of the ancient architecture of the Medzhybizh Fortress in Ukraine under a horizon of debris and soil up to 3 m thick. Deiana and Previato (2023) state GPR and electrical resistivity tomography data are of great interest for the reconstruction of the architectural form of the Roman theatre of Padua and for the definition of its dimensions. The archaeological information obtained with the aid of set of geophysical techniques by Bárta *et al*. (2020) in the historical center of Prague proved to be beneficial even when measurement results were affected by presence of engineering networks, transportation and field obstacles and under conditions when contrast of physical properties between searched objects and their surrounding environment is small.

This work is the first step towards rediscovery of foundations of the Epiphany Cathedral of Brotherhood Monastery in Kyiv. The GPR surveys were aimed at precise positioning of the Cathedral and further planning of archaeological excavation campaign. Professionally performed and scientifically based designation of the location of the lost Cathedral, conservation of architectural and archaeological fragments, accompanied by the wide distribution of the results of on-site research, would restore public interest in the achievements of the past, contribute to heritage preservation and honoring of historical memory of Ukrainian nation ( Goncharova, 2013; Onyshchenko, 2004).

## Methods 

### History and significance of the site

The Kyiv Epiphany Brotherhood Monastery (2 Skovorody St., Kyiv, Ukraine) was founded after 1615 and soon a wooden Epiphany Church was built there, being finished till 1621 ( Kilesso, 2002; Zatyliuk, 2021). Description of the Church, Paul of Aleppo left after his wanderings in 1653, gives a characteristic of the architectural image of the structure in the middle of the 17 century ( Aleppskii, 1897): "There is an arcade around the large church, it has three doors, it has three domes. It is grand, large and has a pulpit with stairs. There is also a wooden round platform in the choir; standing places go in rows to the right and left and face east, in front of them to the right of the choir is a beautiful bishop's place, the back side of which is latticed”.

**Figure 1.**
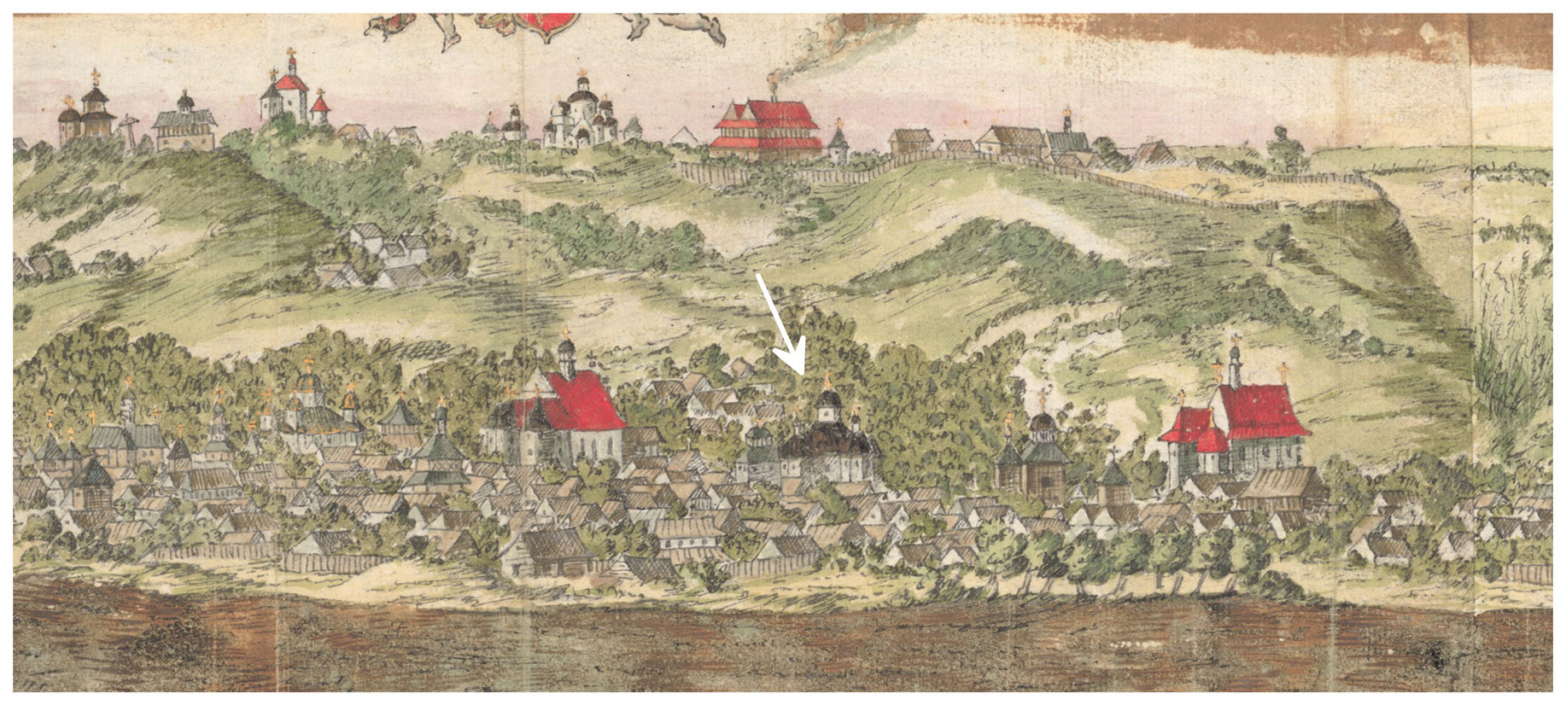
The wooden Epiphany Church suspectedly identified on panoramic drawings of Kyiv created in 1651 by Abraham Westerfeld. Reproduced with permission from ( Panoramic drawing of Kyiv, 1651).

In 1651, another contemporary of Paul of Aleppo, the Dutchman Abraham Westerfeld created panoramic drawings of Kyiv, which are known for the publication of their late replica copies of the end of the 18th century by Yakov Smirnov in 1908 ( Smirnov, 1908). In 2013, Ukrainian historians Bohdan Berezenko and Yuriy Mytsyk reported on a watercolor drawing signed by Westerfeld himself, which was found in the handwritten diary of an anonymous participant in the 1651 war campaign ( Mytsyk & Berezenko, 2013; Panoramic drawing of Kyiv…, 1651). In this picture, the Epiphany Church is suspectedly identified: a large building with a brown roof between two Catholic churches with red roofs. ( Figure 1).

**Figure 2.**
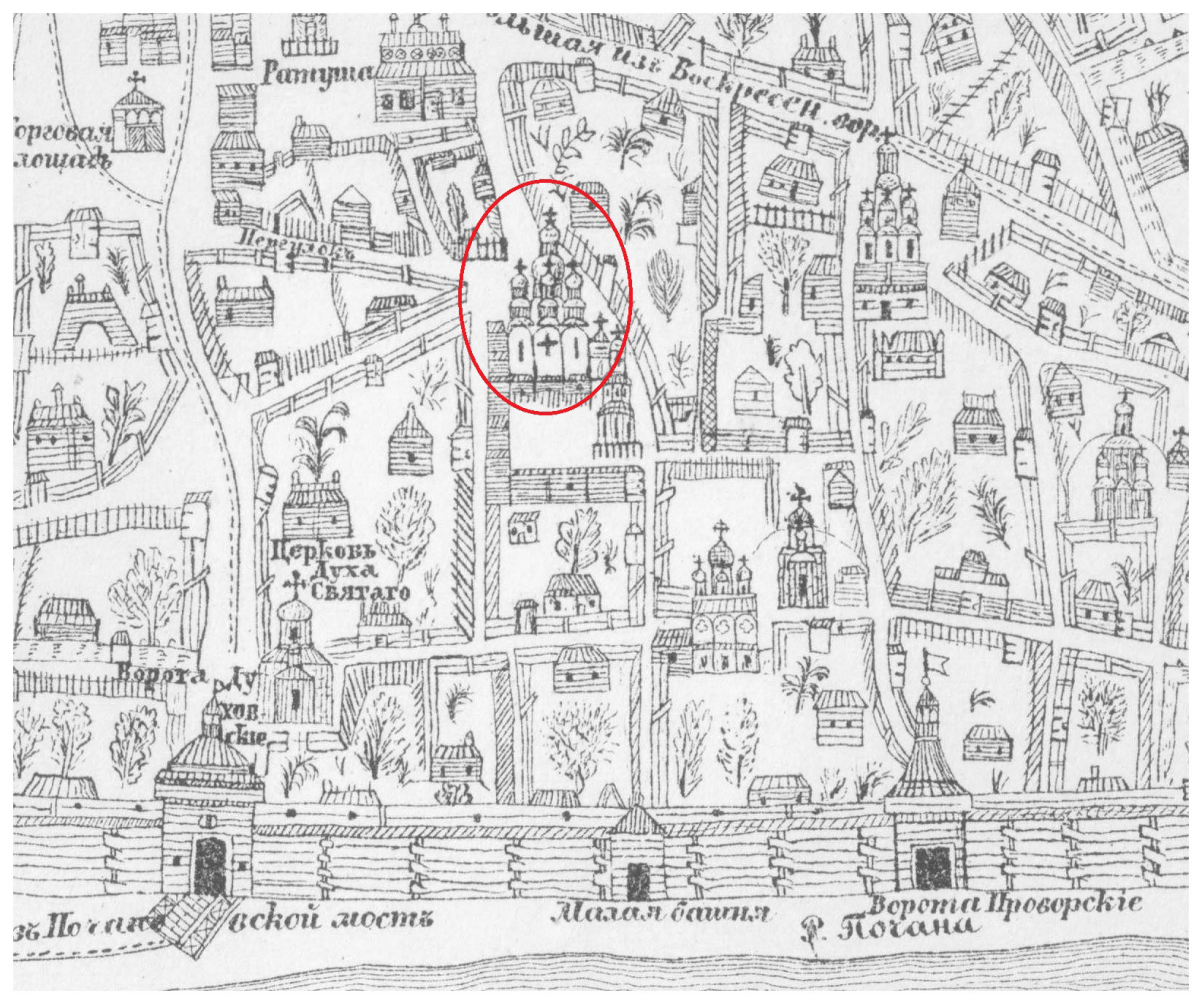
The stone Epiphany Cathedral on a plan of Kyiv created by Ivan Ushakov in 1695. Drawing of a fragment of the plan adapted from ( Plan of Kiev…, 1893). Permission is not needed.

During the 17 century the Church repeatedly suffered from fires. In 1690, the wooden building was dismantled and stone Epiphany Cathedral was constructed at its place ( Holubiev, 1904; Yaremenko, 2017). The first cartographic image of the Cathedral can be found on the plan of Kyiv created by lieutenant colonel Ivan Ushakov in 1695 ( Figure 2) ( Plan of Kiev…, 1893). According to this plan, the Cathedral dominates the architectural landscape of the Kyiv Brotherhood Monastery and the entire lower town of Kyiv named Podil.

**Figure 3.**
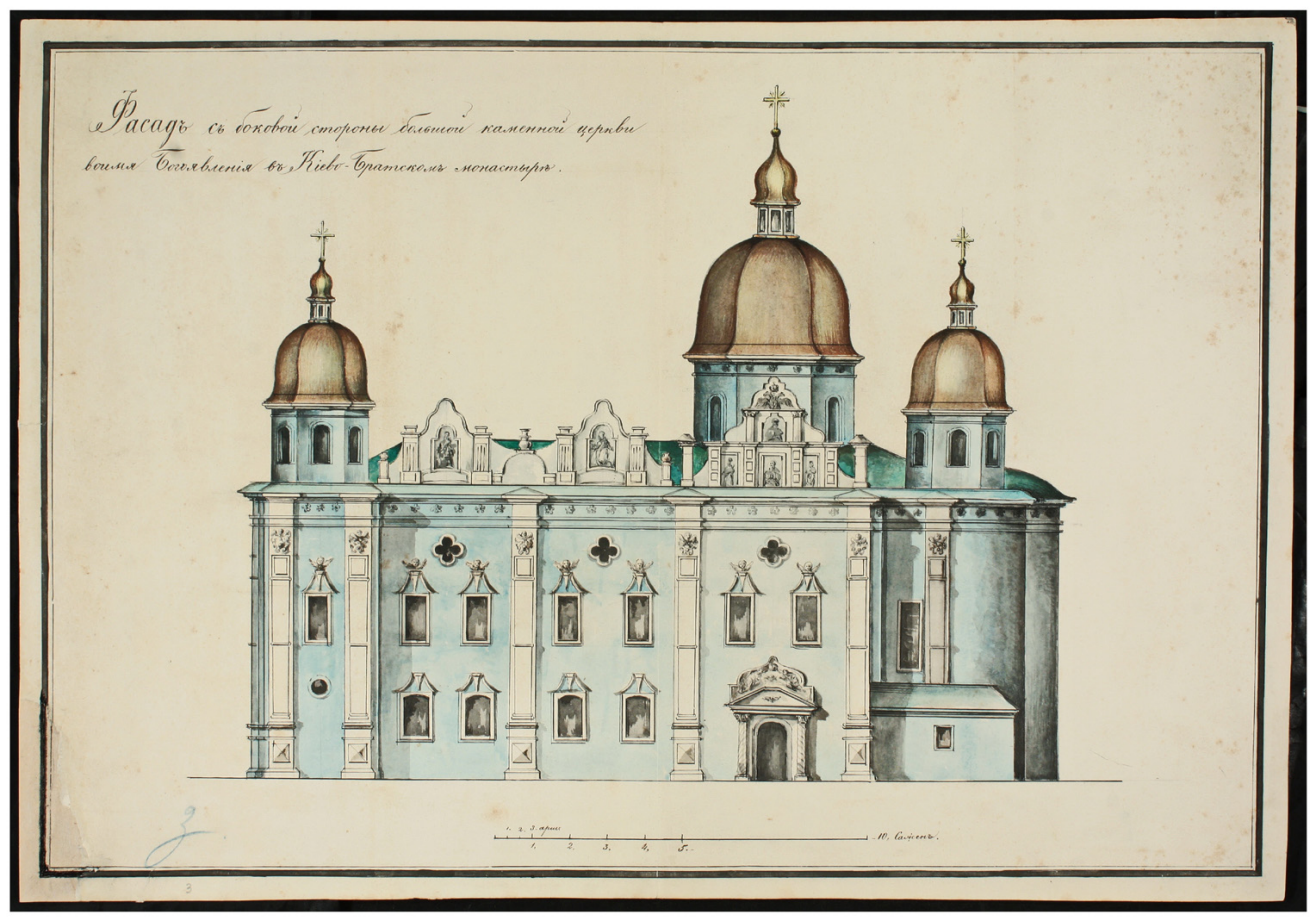
The southern side façade of the Epiphany Cathedral drawn by Andrei Melenskyi in 1825. Reproduced with permission from Institute of Manuscripts of the Vernadsky National Library of Ukraine (IM VNLU), f.28, № 940.

**Figure 4.**
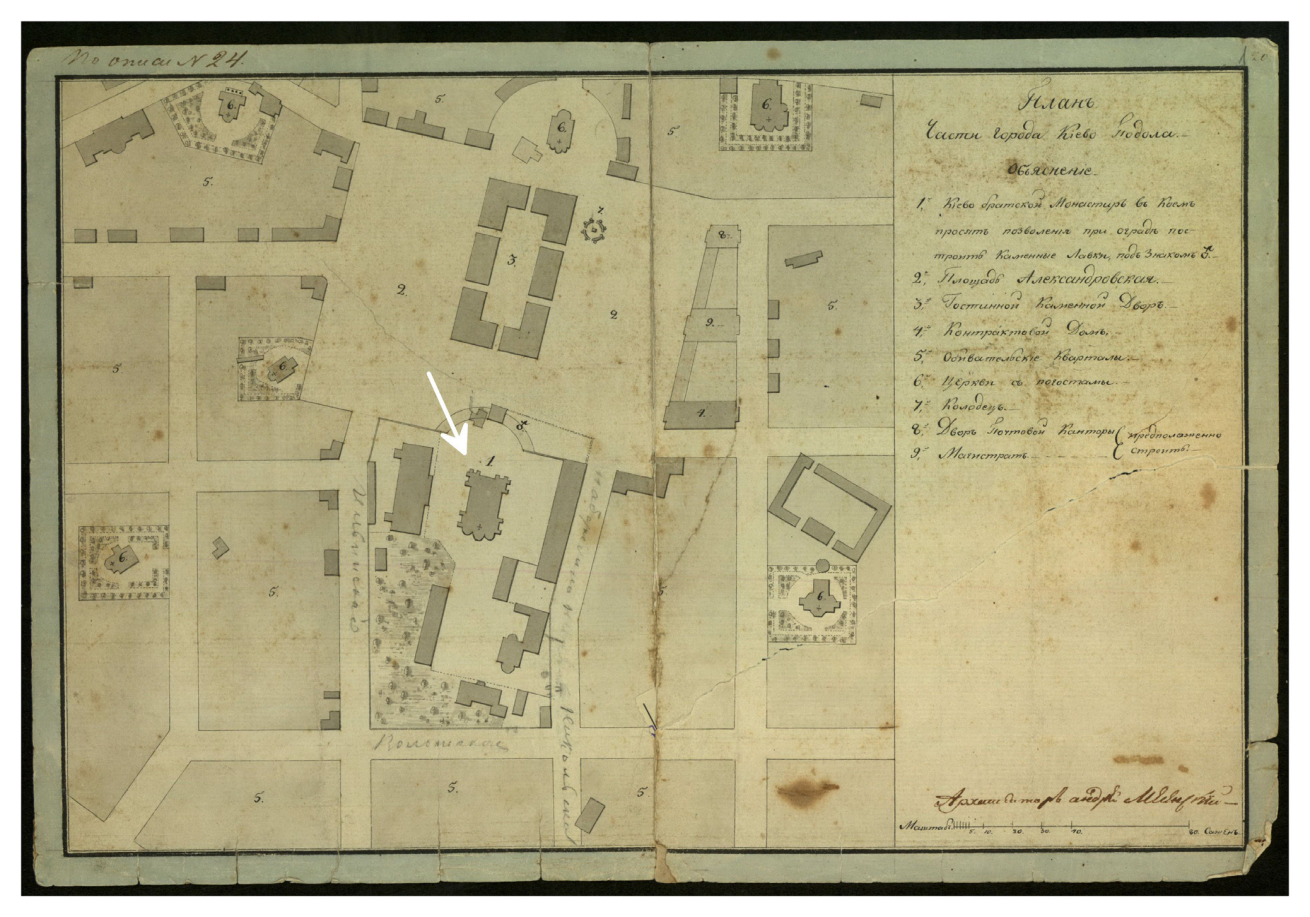
The Epiphany Cathedral and other buildings of Kyiv Brotherhood Monastery on the plan of Podil created by Andrei Melenskyi. Reproduced with permission from IM VNLU, f.28, № 942.

**Figure 5.**
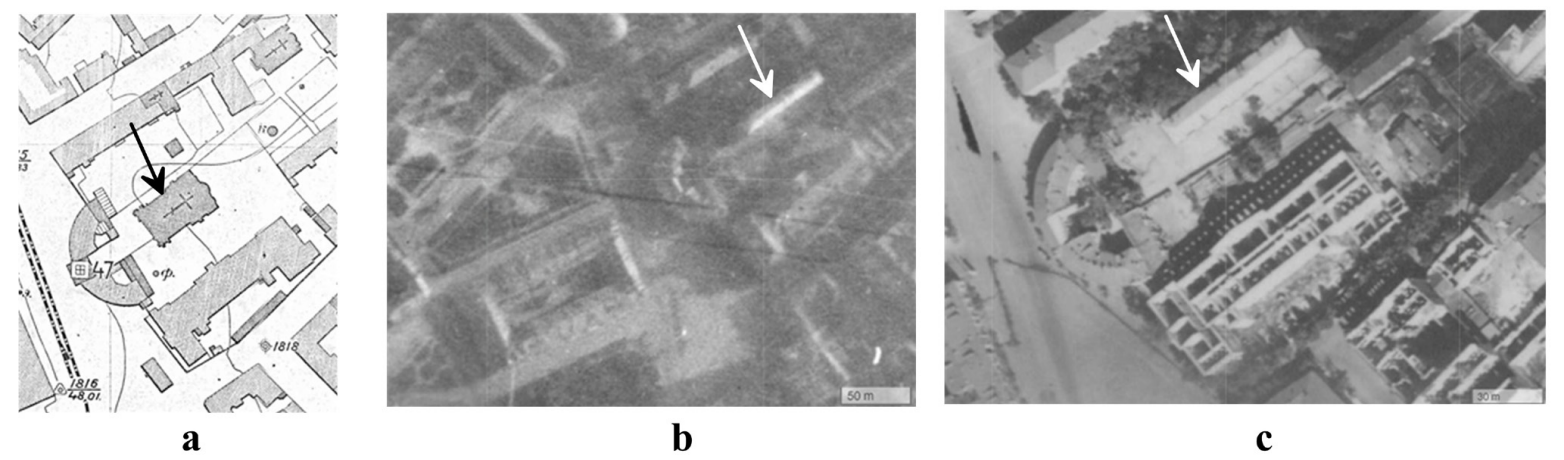
Selected materials that illustrate the architectural situation in the courtyard of Kyiv Brotherhood Monastery in the 1920s–40s: **a** - The Epiphany Cathedral on the topographical survey of the Kyiv City Commune Division in 1923–25; **b** – building 2, constructed on the site of the destroyed Cathedral, on a German aerial photograph of July 3, 1941, US National Archives and Record Administration, Mission GX 506, Film 438, frames 033, 034, 035; **c** - building 2 on the German aerial photograph of September 8, 1943, US National Archives and Record Administration, Mission TUGX 1078, Film 179, frames 118, 119, 120. Images are adapted with permission from Borys Paton State Polytechnic Museum ( https://museum.kpi.ua/map/?d=kyiv&l1=1924.SU.TOPO&l2=&z=11&lon=30.500000&lat=50.45000).

The stone cathedral had been badly damaged by a devastating fire in 1811 and was completely restored in its old place by the architect Andrei Melenskyi ( Figure 3, Figure 4). In this form, the Сathedral existed until 1936, when it was destroyed by the Soviet authorities ( Kilesso, 2002; Kovalynskyi, 2011). Soon after, a four-story building of the headquarters of the Dnipro military flotilla was erected on the foundations of the Epiphany Cathedral, which appeared on German maps and aerial photographs of 1941–43 ( Figure 5). Later, and until 1992, the building belonged to the Kyiv Higher Naval Political School, now it is the 2nd building of the National University "Kyiv-Mohyla Academy" (NaUKMA).

The monastery cemetery was located between the cathedral and the bell tower, while the abbots of the monastery were buried in the cathedral.

**Figure 6.**
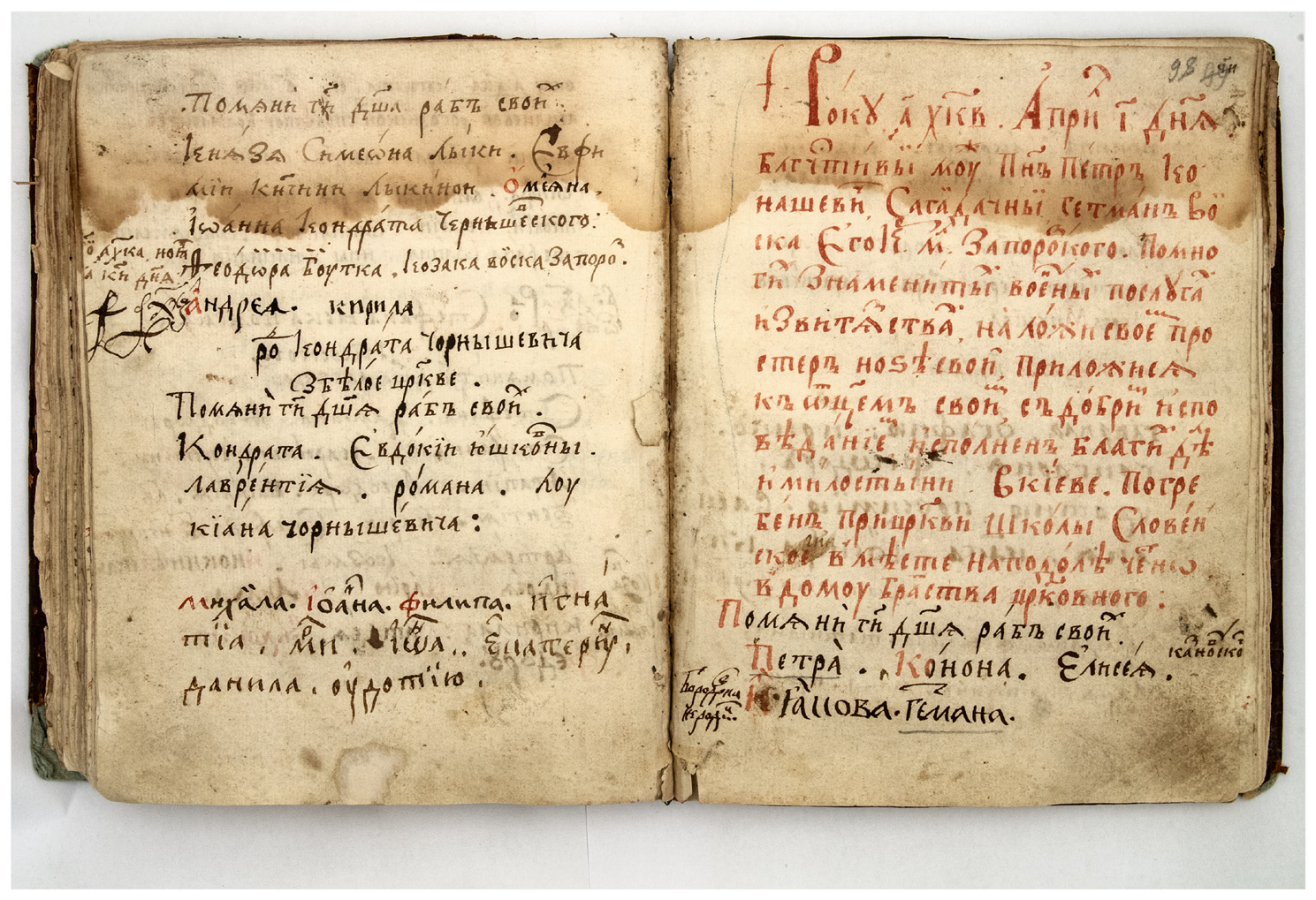
Photograph of the record on death and burial of Hetman Petro Sahaidachnyi in the memorial book of the St. Michael's Golden-Domed Monastery. The record was done with vermillion pigment. Reproduced with permission from IM VNLU, f. 307, №537/1743.

The wooden Epiphany Church is considered to be the burial place of Hetman Petro Sahaidachnyi as recorded in the memorial book of the St. Michael's Golden-Domed Monastery first published by historian Mykhailo Maksimovich ( Maksimovich, 1867) in 1867: "On April 10, 1622, the pious man mister Petro Konashevych-Sagaydachnyi, Hetman of his Royal Grace Zaporizhian Host, after many famous military merits and victories, put his legs on his bed, joined his father with a good confession full of good deeds and mercy in Kyiv. He was buried at the church of the Slovenska school, in the place, in Podil, honestly, in the house of the church brotherhood" ( Figure 6). In addition, in 1678, Paisios Ligarides, Orthodox Metropolitan of Gaza, died in the Kyiv Brotherhood monastery and was buried, as it is believed, in the wooden Church ( Chentsova, 2021).

It is not known for certain whether both burials were moved to the stone cathedral after its construction. However, an interesting version is presented in the article by Volodymyr Burega (2017), where he introduced the work of P. Kudryavtsev "About some burials under the floor of the Great Church of the Brotherhood Monastery". This manuscript tells the story of the discovery of two burials under the floor of the cathedral during its renovation in 1878–1879. According to Kudryavtsev's informant, an unknown connoisseur of antiquities, Antonii Barvynskyi, then Abbot of the Monastery, went down to the underground of the Cathedral, and discovered two coffins there. He opened one of them and found deceased in mitre, the other stone coffin he did not touch. He attributed the first burial to Metropolitan Paisios Ligarides, a famous church figure of the 17th century, and the stone coffin could have belonged to Hetman Petro Sahaidachnyi. While the belonging of the burials to these historical figures causes reasonable doubts among specialists ( Chentsova, 2021), the presence of underground rooms in the cathedral is documented.

Vitalii Kovalynskyi (2011) discovered in the Central State Archives of Public Organizations of Ukraine the resolution of the Presidium of the Kyiv City Council dated March 4, 1935, on reallocation of the Epiphany cathedral for a dormitory of the construction inspection. However, already on March 6, 1935, the regional department of public education informed the Presidium that the construction inspection violated the conditions under which the building was allocated to it. It turned out that "carpenter workshops were functioned here <...>; the iconostasis and side kiots, which have historical and artistic significance, are not surrounded by special fences; workers smoke cigarettes, which threatens fire; the construction inspection *opened the crypts under the church without any permission and opened the coffins*, that makes a criminal offense… ”.

### Past archaeological excavations and observations

**Figure 7.**
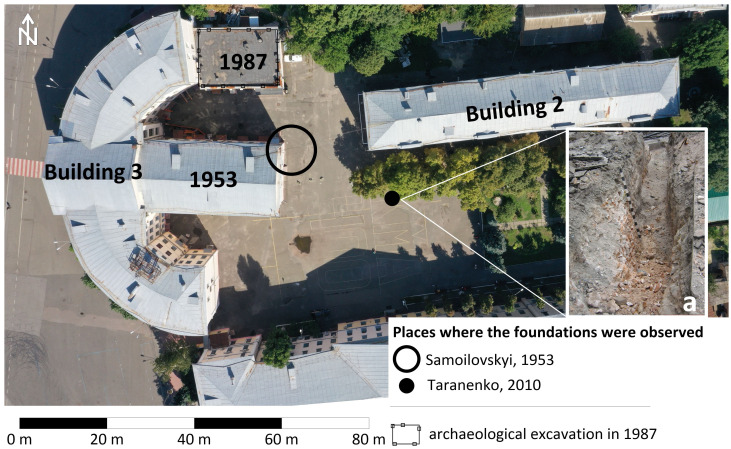
The scheme of placing of archaeological observations and excavations on the site of the Epiphany Cathedral; **а** - remains of the Cathedral`s foundation photographed in 2010 by S. Taranenko, image after Taranenko (2013). Permission is not needed.

During the Soviet and post-Soviet times, the foundations of the Epiphany Cathedral were twice documented by archaeologists. In 1953, Illya Samoilovskyi (1953) reported the following observation: "In the estate of the former Brotherhood Monastery, during construction, in a wide pit 4 m deep, laid on the site of the Epiphany Cathedral, its foundation was discovered in the northeastern part of the pit. The foundation reached 2 m deep from the present surface. Near the foundation, two oak coffins with burials in them were found, which were destroyed by an excavator". Obviously, this paragraph discusses the construction of an extension to the semi-circular building, currently 3rd building of NaUKMA. The foundations of the cathedral were discovered by I. Samoilovsky in the northeastern part of the construction pit of the future extension ( Figure 7).

For the second time, the foundations of the stone cathedral were partially uncovered in the recent past, on August 31, 2010, during earthworks for the repair of heating networks in the yard of NaUKMA. At the repair site, archaeologist Serhyi Taranenko (2013) discovered a strong brick foundation that reached a depth of more than 2 m, covered with a half-meter layer of mixed soil and construction debris. ( Figure 7).

It is worth mentioning that in 1987, the Podil expedition of the Institute of Archeology of the National Academy of Sciences excavated a part of a cemetery functioning from the late Middle Ages until the 19th century to the northwest of the foundations of the cathedral ( Sahaidak *et al*., 1995). Authors unearthed 12 burials, the grave pits reached 2.2–3.15 m from the level of the modern surface. The burials were discovered while digging a pit for construction of another extension to a semi-circular building 3 of NaUKMA. The foundations of the Cathedral, however, were not revealed by this excavation ( Figure 7).

### Acquisition and processing of the GPR data

GPR has been used to search for the foundations of Epiphany Cathedral. A challenge to survey the site using this technique can be related to: 1) presence of city communications (electrical cables, water and heating supply pipes) crossing the site; 2) limitations of the propagation of the electromagnetic energy in clay-rich and highly conductive soil ( Conyers, 2017). The presence of construction remnants, uneven leveling of the yard, and numerous old utilities, both functional and non-functional, created a complex subsurface environment. Repeated repairs to utilities further complicated the subsurface structure. Moreover, water leakage from the sewer system can significantly affect the penetration depth of the GPR as water tends to absorb the radar waves.

**Figure 8.**
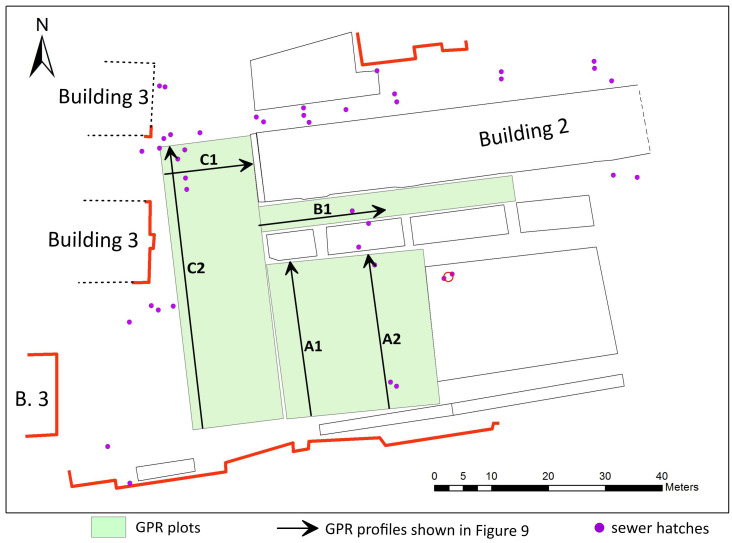
Topographic plan of the site demonstrating location of geophysical survey plots in the courtyard of NaUKMA.

Measurements were performed within three rectangular plots georeferenced using tacheometer Total Station/Trimble M3 5‘ ( Figure 8). The GPR prospecting was carried out with a VIY-5-37 (Transient technologies LLC, Ukraine) instrument with equipped with ground coupled shielded antennas with nominal middle frequencies of the emitted EM wave at 300 and 700 MHz. Data were acquired in continuous mode along 0.5-m spaced survey lines in both perpendicular directions, using 500 samples per trace, 240 ns time range and constant sampling interval of 32mm along the inline direction. The data were subsequently processed using standard two-dimensional processing techniques by means of the Synchro3 software ( http://viy.ua/e/software/synchro.htm).

The processing flow-chart consists of the following steps: (I) zero level setting-to determine the depth correctly, it is necessary to match the beginning of the depth scale with a certain point of the direct pulse (e.g. maximum amplitude); (II) wavelet filtering in order to suppress effectively low-frequency fluctuations and high-frequency noise; (II) the windowed background removal tool subtracts an averaged trace from each trace of the profile, with the width of the window for averaging specified by the total number of traces; (III) manual gain, to adjust the acquisition gain function and enhance the visibility of deeper anomalies. By means of the Planner software, the radargrams were subsequently merged together into three-dimensional volumes and visualized in various ways in order to enhance the spatial correlations of anomalies of interest. The average electromagnetic wave velocity was estimated as 100 m/µs from the known depth of water pipe hyperbola located at the site ( Conyers, 2017).

## Results

### The GPR results

**Figure 9.**
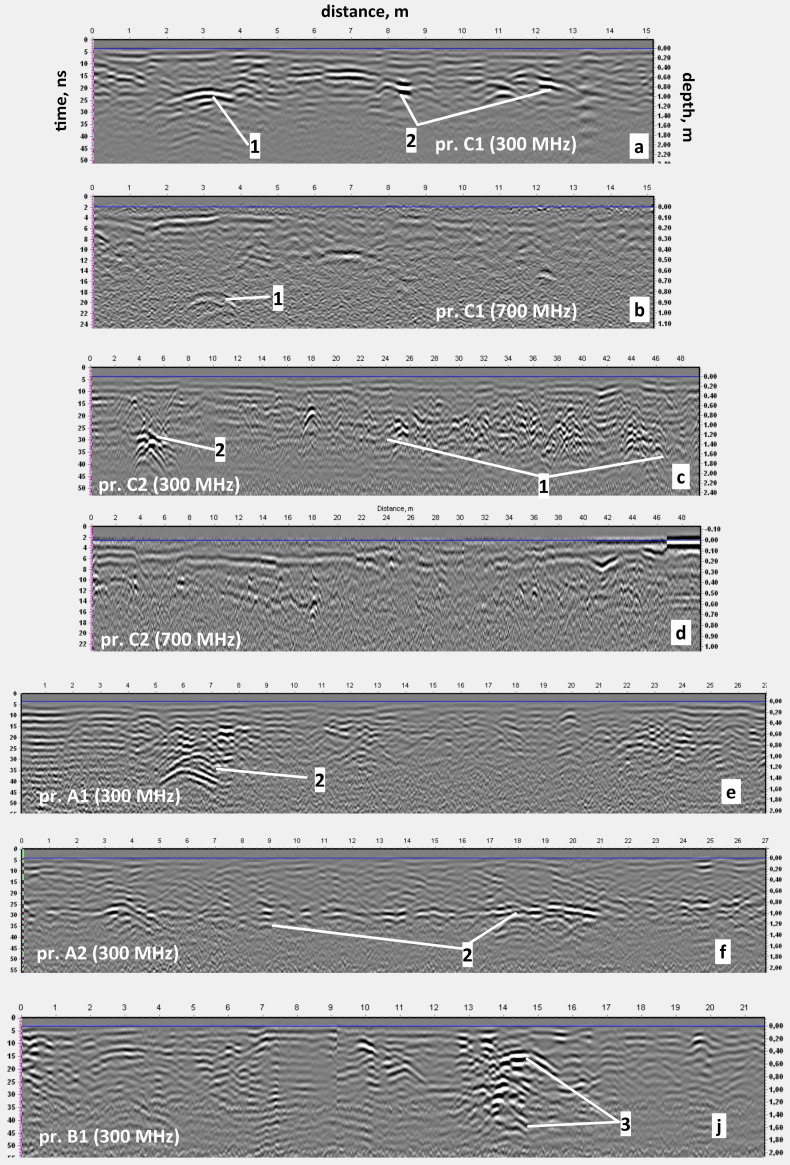
Examples of radargrams with characteristic anomalies caused by: 1 - the foundation of the Cathedral; 2 – heating line; 3 – technical pit. The positions of the corresponding GPR profiles are shown in Figure 8.

In the time interval of 15–50 ns, a number of local anomalies of the electromagnetic signal were recorded ( Figure 9). As already mentioned, GPR prospection is significantly hampered by an area's clay-rich geology and heterogeneous subsurface structure. From profiles recorded with the 700 MHz antenna subsurface features can hardly be identified since such a frequency does not provide enough penetration depth ( Figure 9 b, d).

Urban communications can easily be interpreted from 300 MHz radargrams and C-scans (horizontal sections) by strong hyperbola-shaped reflections repeating on neighboring profiles and thus forming characteristic linear structures. In particular, Figure 9 a, c, e, j presents examples of such radargram. Figure 9 f illustrates the case when the GPR profile stretches along a concrete box with a water pipe.

The C-scans were compared with existing maps of urban communications on the courtyard of NaUKMA. In this way, it was possible to clarify sources of various anomalies - concrete boxes of heat networks, water supply pipes, technical underground structures, etc. - and to discover new features. This made it easier to identify reflections possibly related to the foundations of the Cathedral.

**Figure 10.**
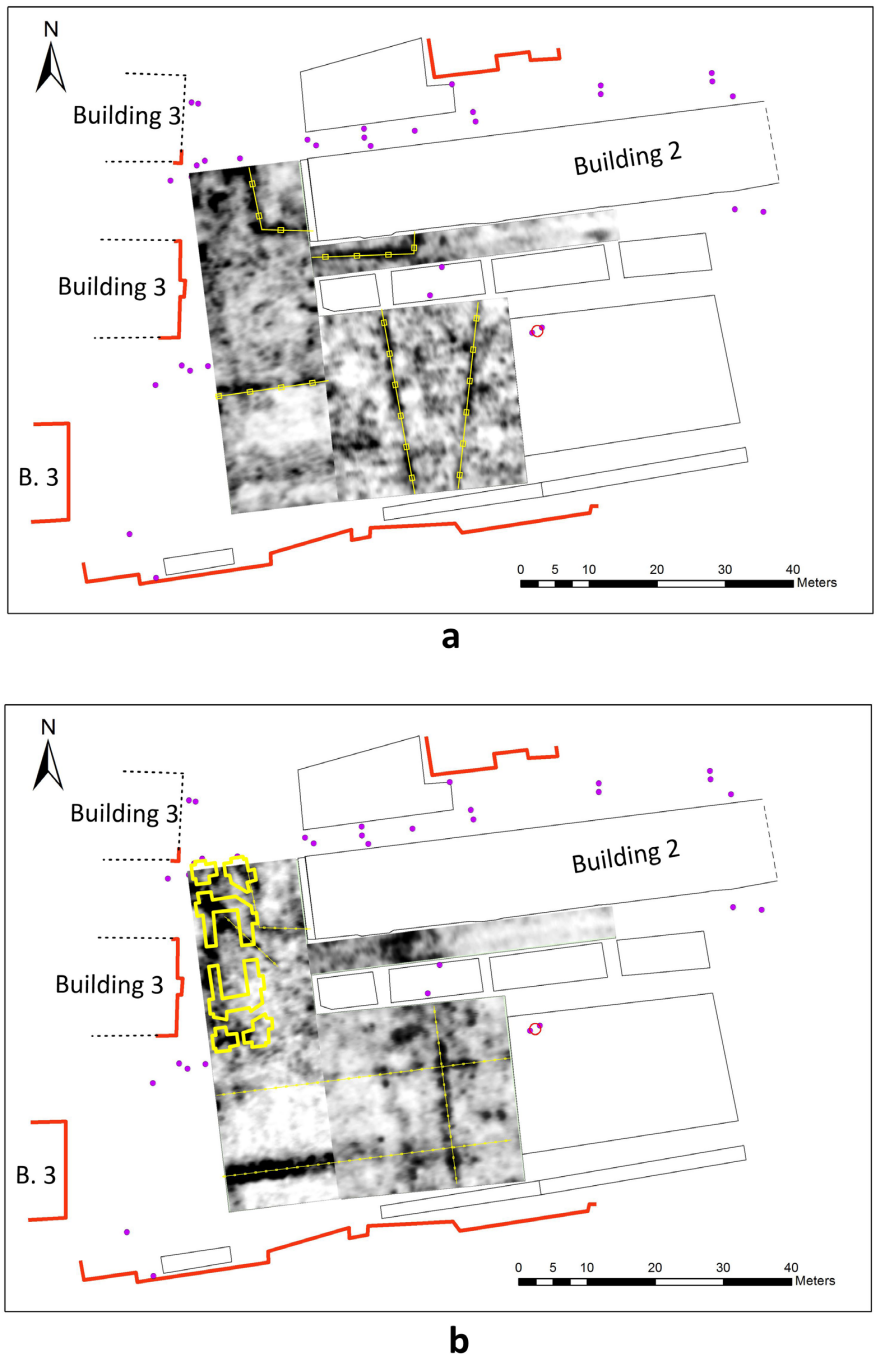
GPR C-scans (horizontal sections) of the courtyard of the Kyiv-Mohyla Academy: **a** - depth 0.5–0.7 m; **b** - depth of 0.7–0.9 m. Urban communications and fragments of the foundation of the Epiphany Cathedral interpreted from GPR results are marked with yellow.

Figure 10 presents C-scans from depths of 0.5–0.7 m and 0.7–0.9 m. Urban communications of various purposes are laid at different depths and often intersect. On the C-scan from the depth range of 0.7–0.9 m, we can see a linear zone of reflections, which can be attributed to the remains of the western massive wall of the Cathedral. The examples of reflections originating from the wall are shown on the 300 and 700 MHz radargrams on Figure 9 a and b. On the 300 MHz profile C2 ( Figure 9 c) the wall appears as a series of diffractions and does not show up at 700 MHz radargram ( Figure 9 d).

### Reconstruction of location and archaeological proof

**Figure 11.**
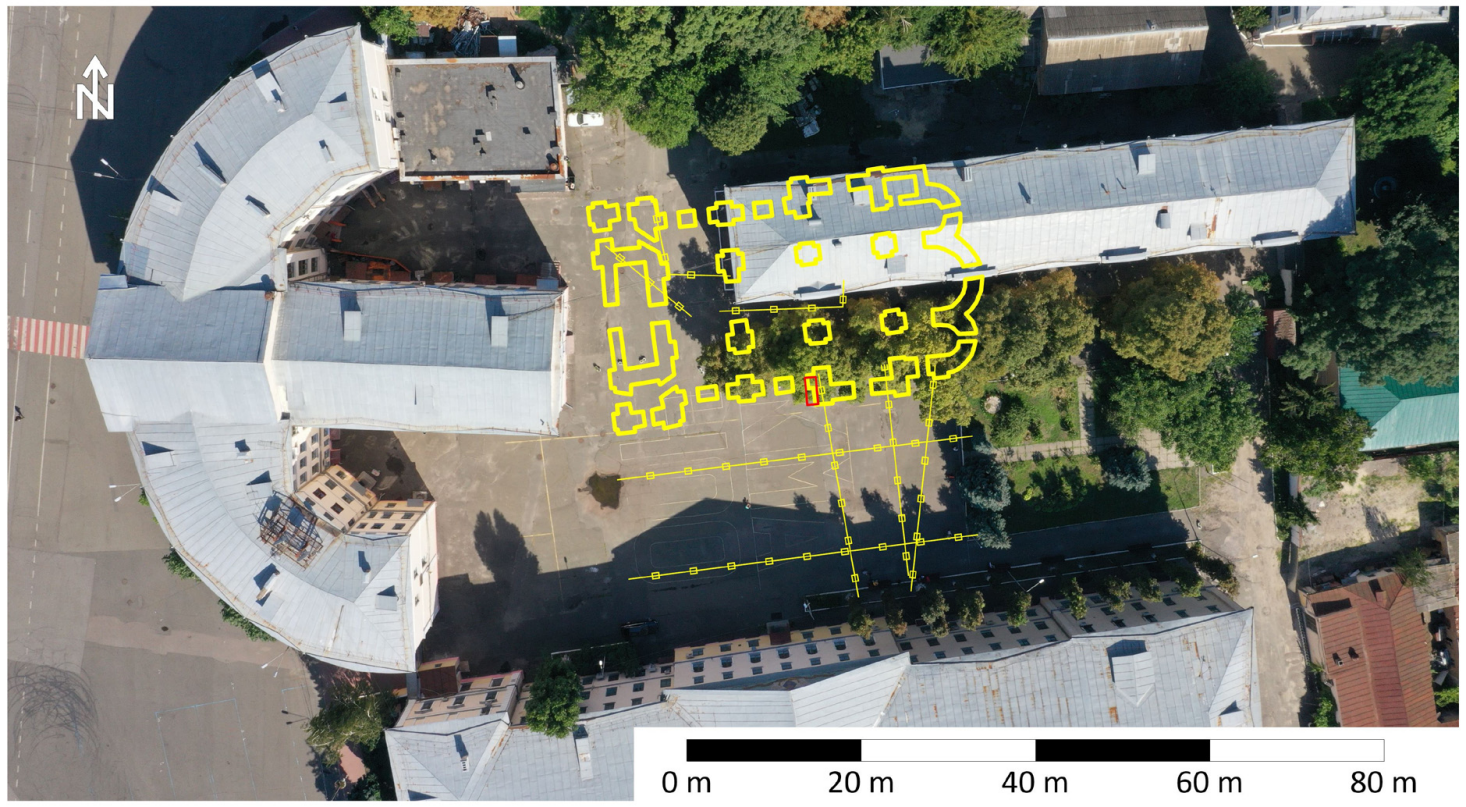
Reconstruction of location of the Epiphany Cathedral on the courtyard of modern Kyiv-Mohyla Academy. The test trench is marked with a red rectangle. Depiction of foundations is retrieved from a book of Ivan Funduklei (1847). Permission is not needed.

Based on the defined location of separate parts of the foundation and known size and shape of the entire structure ( Funduklei, 1847), it is possible to restore the placement of the Epiphany Cathedral of Kyiv Brotherhood Monastery, represented on Figure 11. The western wall of the Cathedral lays 5m to the east from the extension of 1953 to the semi-circular building 3 of NaUKMA. The building 2 stays directly on the northeast quarter of the Сathedral. The outhern half of it is free from modern structures.

The reconstruction shows that informative archaeological excavations can be conducted without destroying modern buildings, using controlled excavation techniques. This is a case when remains of famous landmark and modern buildings can coexist. Foundations of the Epiphany Cathedral can be integrated into the design of NaUKMA architectural ensemble, preserved and made accessible to the public. The majority of underground structures suspected in the nave of the destroyed cathedral also could be investigated. Including the place indicated by Kudryavtsev ( Burega, 2017) as the alleged location of Hetman Sahaidachnyi`s burail (near the right central pillar). Archaeological excavations of the remains of the Epiphany Cathedral can indeed hold exceptional importance for the people of Ukraine, particularly during challenging times such as the struggle against Russian aggression.

In 2022 the site with the remains of the Epiphany Cathedral was certified as a monument of history, archaeology and architecture in the Register of cultural heritage of Kyiv. In 2023 it had been added to the State Register of Tangible Monuments of Ukraine, that opened up opportunities for continued archaeological research.

**Figure 12.**
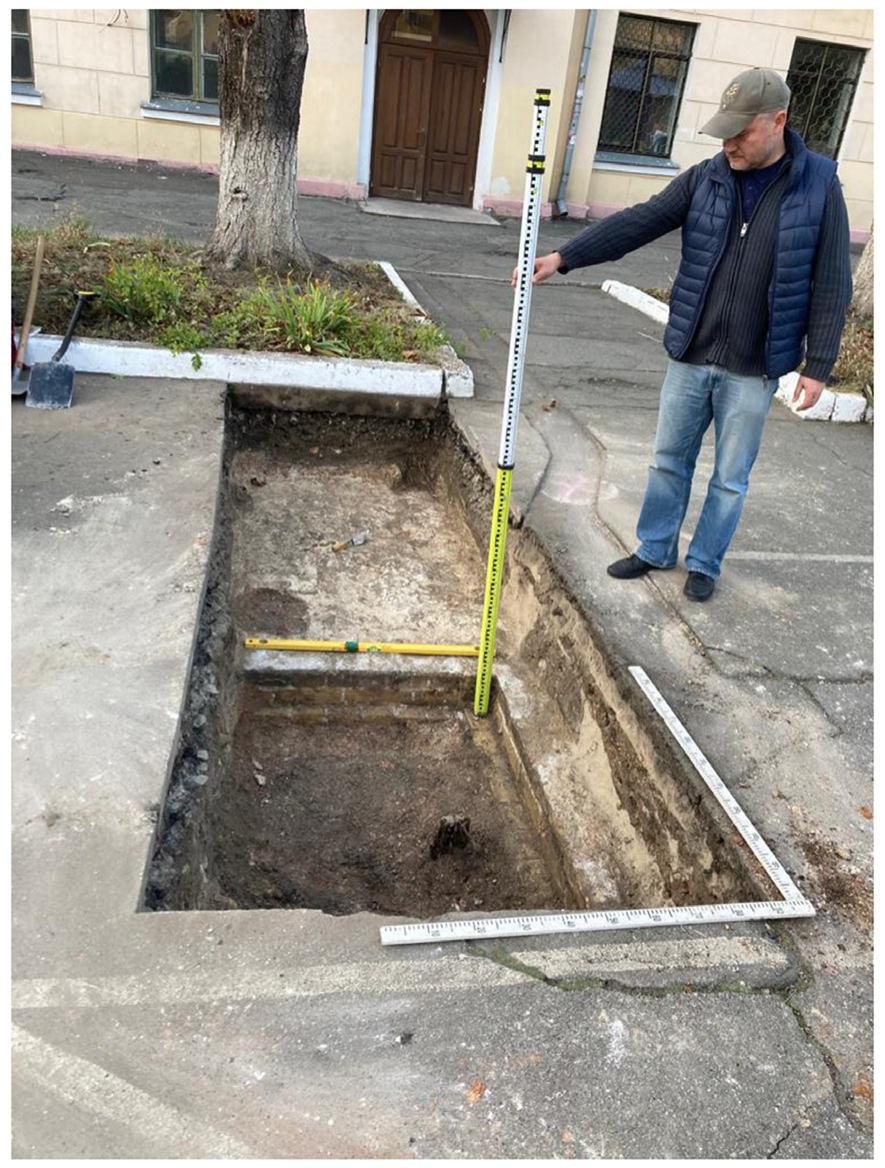
Photograph of the test trench showing an unearthed corner between the southern wall and the transept of the Epiphany Cathedral.

The proposed reconstruction has been used to put the test archaeological trench. In October 2023 an excavation with an area of 4x1 m opened the corner made by southern wall and transept of the Cathedral ( Figure 12). The discovery of this unique architectural detail makes it possible to unambiguously tie the Cathedral to the place. Further archaeological excavations are to be continued in 2024.

The territory of the former Kyiv Brotherhood Monastery was practically not disturbed by any significant construction in the 17th–21st centuries. Therefore, cultural layer there has preserved its intact archaeological stratigraphy, making this site a unique source in the history of Kyiv Podil since Ancient Rus time. Its` systematic archaeological research should begin with a complex of non-invasive geophysical studies.

## Conclusions

A comprehensive understanding of the significance and archaeological potential of the site of destroyed Epiphany Cathedral of Kyiv Brotherhood Monastery was established by combining historical research with ground penetrating radar technique. The historical research provides context and background information about the Cathedral, including its construction, renovations, previous uses, and any known archaeological discoveries. Two locations of Cathedral`s foundations were identified as well as excavated area nearby, which did not reveal remains of the structure.

The analytical approach implemented in this paper allowed us to precisely target GPR measurements. In this study, a dual-frequency GPR system (300 MHz and 700 MHz) was used. The 300 MHz antenna proved to be effective in penetrating clay-rich cultural layers studied at a depth of 2.0 m. This allowed us to image and separate the city communications and fragments of the foundation in the studied area in the radargrams and C-scans. The ground-penetrating radar survey showed the best preservation of the foundations of the massive western wall of the Cathedral.

The proposed reconstruction of the location of Cathedral`s foundation is to be used in the development of archaeological investigation, conservation and management plans for the site, ensuring its protection and preservation.

We believe this study facilitates the use of non-invasive techniques in the cultural resources management by advancing the field of urban geophysical prospection in Ukraine.

## Data Availability

Zenodo. Data repository of GPR survey of the Epithany Cathedral of Kyiv Brotherhood Monastery by Kseniia Bondar. DOI: 10.5281/zenodo.8302838 This project contains the following underlying data: GPRData-EpiphanyCathedralKyiv (The folder contains raw GPR survey data as separate Planner projects for plots measured in different directions). Data are available under the terms of the Creative Commons Attribution 4.0 International license (CC-BY 4.0).
