# Supplementary material for: Ground-penetrating radar scanning and historical interpretation of the location of the destroyed Epiphany Cathedral in Kyiv Brotherhood Monastery (Ukraine)
Source: Open Res Eur. 2024 Jun 24;3:196. Originally published 2023 Nov 14. [Version 2] doi: 10.12688/openreseurope.16592.2 (PMC11153991; doi:10.12688/openreseurope.16592.2)
Supplement: Peer review history

# Reviewer report: Reviewer response for version 2

**DOI:** 10.21956/openreseurope.19545.r41605

**Published:** 2024-07-17

**Authors:** Caglayan Balkaya

**Affiliations:** 1 Department of Geophysical Engineering, Süleyman Demirel University, Isparta, Turkey

In this revised manuscript, the authors have addressed the points of criticism and also presented the results of a test excavation that served to validate the geophysical results. The conclusions presented in the paper are effectively underpinned by the ground-penetrating radar (GPR) results, detailed explanations and the provision of supporting historical and archaeological evidence. Given the satisfactory manner in which the authors have addressed the concerns raised during the review process in their revised manuscript, I am satisfied with the revised version.

If any, are all the source data and materials underlying the results available?

Yes

Is the work original in terms of material and argument?

Yes

Is the work clearly and cogently presented?

Yes

Is the argument persuasive and supported by evidence?

Yes

Does the research article contribute to the cultural, historical, social understanding of the field?

Yes

Does it sufficiently engage with relevant methodologies and secondary literature on the topic?

Yes

Reviewer Expertise:

Exploration and computational geophysics

I confirm that I have read this submission and believe that I have an appropriate level of expertise to confirm that it is of an acceptable scientific standard.

# Reviewer report: Reviewer response for version 2

**DOI:** 10.21956/openreseurope.19545.r41606

**Published:** 2024-07-08

**Authors:** Mercedes Solla

**Affiliations:** 1 Universidade de Vigo, Vigo, Galicia, Spain

The comments and revisions made from my first review have not been addressed.

If any, are all the source data and materials underlying the results available?

Yes

Is the work original in terms of material and argument?

Yes

Is the work clearly and cogently presented?

Yes

Is the argument persuasive and supported by evidence?

Yes

Does the research article contribute to the cultural, historical, social understanding of the field?

Yes

Does it sufficiently engage with relevant methodologies and secondary literature on the topic?

Partly

Reviewer Expertise:

Ground-penetrating radar, cultural heritage prospection, civil engineering

I confirm that I have read this submission and believe that I have an appropriate level of expertise to confirm that it is of an acceptable scientific standard, however I have significant reservations, as outlined above.

# Reviewer report: Reviewer response for version 2

**DOI:** 10.21956/openreseurope.19545.r41608

**Published:** 2024-07-02

**Authors:** Vicente Bayarri

**Affiliations:** 1 Universidad Europea del Atlantico, Santander, Cantabria, Spain

The article focuses on the application of ground-penetrating radar (GPR) with historical research to locate and identify the remains of the Epiphany Cathedral of the Kyiv Brotherhood Monastery.

 The goal is to provide a basis for the conservation and archaeological investigation of the site, which aligns with the journal's goals of advancing archaeological prospection and site characterization.

 I will now go on to answer the requested questions:

 1. Is the work clearly and accurately presented and does it cite the current literature?

 The work is accurately presented, providing detailed results of the GPR survey, the interpretation of anomalies, and the archaeological significance of the findings. It now cites a range of current literature. Integrating historical data with geophysical survey results is positive.

 2. Is the study design appropriate and does the work have academic merit?

 The study design is appropriate to the goals, using suitable methodologies and integrating historical data effectively. The work has academic merit, contributing to both the field of archaeology and the preservation of cultural heritage. It stands as a example of how modern technology can be used with historical research to uncover and preserve the past.

 3. Are sufficient details of methods and analysis provided to allow replication by others?

 The paper's detailed methodology, including equipment specifications, data acquisition parameters, and data processing techniques, provides sufficient details for replication by other researchers.

 4. If applicable, is the statistical analysis and its interpretation appropriate?

 The paper does not include formal statistical analysis, but the interpretation of GPR data is conducted methodically and appropriately. The technical explanations regarding the use of different frequencies and the correlation with existing urban maps provide a robust framework for understanding the subsurface features

 5. Are all the source data underlying the results available to ensure full reproducibility?

 The study provides sufficient information to allow replication by others.

 6. Are the conclusions drawn adequately supported by the results?

 The paper's conclusions are well-supported by the GPR survey results, explanations, and corroborative historical and archaeological evidence. Integrating various data sources and the logical interpretation of findings provide a solid foundation for the conclusions drawn.

If any, are all the source data and materials underlying the results available?

Yes

Is the work original in terms of material and argument?

Yes

Is the work clearly and cogently presented?

Yes

Is the argument persuasive and supported by evidence?

Yes

Does the research article contribute to the cultural, historical, social understanding of the field?

Yes

Does it sufficiently engage with relevant methodologies and secondary literature on the topic?

Partly

Reviewer Expertise:

GPR, data integration, data processing, remote sensing, geomatics

I confirm that I have read this submission and believe that I have an appropriate level of expertise to confirm that it is of an acceptable scientific standard.

# Reviewer report: Reviewer response for version 2

**DOI:** 10.21956/openreseurope.19545.r41607

**Published:** 2024-07-02

**Authors:** Nikos Papadopoulos

**Affiliations:** 1 Foundation for Research and Technology - Hellas, Nikolaou Plastira, Greece

I went through the revised version of the paper. The authors addressed the comments and they also provided the results from a test excavation pit verifying the geophysical results.

 I recommend the indexed of the paper as indicated in the revised version.

If any, are all the source data and materials underlying the results available?

Partly

Is the work original in terms of material and argument?

Yes

Is the work clearly and cogently presented?

Partly

Is the argument persuasive and supported by evidence?

Partly

Does the research article contribute to the cultural, historical, social understanding of the field?

Yes

Does it sufficiently engage with relevant methodologies and secondary literature on the topic?

Yes

Reviewer Expertise:

Applied geophysics, archaeological geophysics, geoinformatics in cultural and natural resources managmentn

I confirm that I have read this submission and believe that I have an appropriate level of expertise to confirm that it is of an acceptable scientific standard.

# Reviewer report: Reviewer response for version 1

**DOI:** 10.21956/openreseurope.17909.r38777

**Published:** 2024-06-05

**Authors:** IŞIK Nursen

**Affiliations:** 1 Architecture, Dicle University, Diyarbakır, Diyarbakır, Turkey; 2 Dicle Universitesi, Diyarbakır, Diyarbakır, Turkey

Dear Editor,

  The research was completed by determining the damages in the structure of Epiphany Cathedral in Kyiv Brotherhood Monastery (Ukraine) using the GPR method and evaluating the data obtained. It is very valuable that in the research article, an original building of Cultural Heritage nature is discussed and the problems in the building are detailed using the GPR method. However, completing some deficiencies in the research will strengthen the research. These;

 • Although the history and development process of the Epiphany Cathedral in Kyiv Brotherhood Monastery (Ukraine) structure is explained, its current situation is not mentioned. These need to be added.

 • Adding a section explaining the structural problems of the historical building will strengthen the research.

 • It was stated in the research that instrumental determinations were made using the GPR method. Introducing the device on which GPR scans are performed (antenna status) will contribute to the study.

 In conclusion; It is thought that the study will contribute to the literature after the regulations.

If any, are all the source data and materials underlying the results available?

Yes

Is the work original in terms of material and argument?

Yes

Is the work clearly and cogently presented?

Yes

Is the argument persuasive and supported by evidence?

Yes

Does the research article contribute to the cultural, historical, social understanding of the field?

Yes

Does it sufficiently engage with relevant methodologies and secondary literature on the topic?

Yes

Reviewer Expertise:

Structural problems in historical buildingsApplicability of non-destructive methods in historical buildingsGPR applications in historical buildingsReinforcement techniques in historical buildings

I confirm that I have read this submission and believe that I have an appropriate level of expertise to confirm that it is of an acceptable scientific standard.

# Reviewer report: Reviewer response for version 1

**DOI:** 10.21956/openreseurope.17909.r36444

**Published:** 2024-05-31

**Authors:** Mercedes Solla

**Affiliations:** 1 Universidade de Vigo, Vigo, Galicia, Spain

This reviewer has read this article with great interest. Although there are numerous GPR works on archaeology published in the literature, this article presents a unique case study (registered as a monument of Ukraine) that deserves to be indexed. Moreover, the data is freely available in Zenodo repository.  However, it needs some minor improvements before accepting it for indexed. 

 My main criticisms are: 

 - In section "Acquisition and processing for the GPR data": Please, check the sentence "The GPR prospecting was carried out with a VIY-5-37 (Transient technologies LLC, Ukraine) instrument with equipped with ground coupled shielded antennas with nominal middle frequencies of the emitted EM wave at 300 and 700 MHz". Please, rewrite the sentence, it is difficult to understand in its current form. 

 - In section "The GPR results": Please, rewrite this sentence for a better understanding "As the reflection is probably caused by the brick foundation we can state, that only the upper edge of it at a depth of 0.7 m is revealed with GPR, but not the bottom (Figure 9 a, b)."

 - In the same section, and sentence "Urban communications can easily be interpreted from radargrams and C-scans (horizontal sections) by strong hyperbola-shaped reflections". This sentence is true but, for non-expert readers in the method, it should be explained why the reflections produced are hyperbolas (or to include some reference). 

 - In the same section, and sentence "Figure 9 d illustrates the case when the GPR profile stretches along a concrete box with a water pipe": Could authors identify the signal polarity inversion due to the presence of water? Please, state in the text. 

 - In conclusions section authors use the term "ground-penetrating radar" while in the rest of the document they have used "ground penetrating radar". Please, be consistent and use the same term in the whole manuscript. 

 - The conclusions section can be improve. For examples, what further investigations/works can be planned?, what limitations for GPR were found? type of soils? attenuation by clay-rich layers?, what about the frequency antenna resolution?.

If any, are all the source data and materials underlying the results available?

Yes

Is the work original in terms of material and argument?

Yes

Is the work clearly and cogently presented?

Yes

Is the argument persuasive and supported by evidence?

Yes

Does the research article contribute to the cultural, historical, social understanding of the field?

Yes

Does it sufficiently engage with relevant methodologies and secondary literature on the topic?

Partly

Reviewer Expertise:

Ground-penetrating radar, cultural heritage prospection, civil engineering

I confirm that I have read this submission and believe that I have an appropriate level of expertise to confirm that it is of an acceptable scientific standard, however I have significant reservations, as outlined above.

# Reviewer report: Reviewer response for version 1

**DOI:** 10.21956/openreseurope.17909.r36255

**Published:** 2024-05-25

**Authors:** Caglayan Balkaya

**Affiliations:** 1 Department of Geophysical Engineering, Süleyman Demirel University, Isparta, Turkey

The article describes the results of a ground penetrating radar (GPR) survey carried out at the Epiphany Cathedral of the Brotherhood Monastery site in Kyiv, Ukraine. This cathedral, which was destroyed by the Soviets in 1936, is known as the burial place of Hetman Petro Sahaidachnyi, a well-known Ukrainian Cossack leader. The study draws on archival documents, plans, blueprints, photographs, and cartographic material dating from the 17th to 19th centuries. The historical data, from 1615 (the beginning of the construction of the wooden Epiphany Church) to the present day, is used to interpret the geophysical results obtained through GPR scans. The historical story of the manuscript is exciting and promising. In its present form, however, it has some weaknesses that should be corrected to benefit the journal’s reader.

1. I think that the use of an antenna system with a center frequency of 700 MHz and a time window of 240 ns in the data acquisition part is unrealistic due to the shortcomings mentioned in the relevant part, which include the presence of urban communication and the presence of a clay-rich conductive medium. The processed radargrams show that the reflections from hidden objects below the surface originate from a depth of about 45-50 ns. The question arises as to why the authors were not able to carry out various test measurements in the investigated area in advance of the study to obtain optimal detection parameter sets
2. The effects of undesired, coherent clutter signals called air waves should be removed from the radargrams.
3. The scale of the processed radargrams should correspond to the measuring lengths in Figure 9.
4.  For the interpretation of the radargrams 9c and 9e, it would be nice to give the reader a velocity from analyzing the discovered hyperbolas. In addition, a fence plot with the radargrams in Figure 9 could increase the attractiveness of the study.
5. It may be helpful to mark the investigated areas on the aerial photograph in Figure 7 and to explain in the text how many GPR scans were performed in these areas and in which direction the measurements were taken during the investigation.
6. The software used to create the depth section maps shown in Figures 10a and 10b should be explained.
7. The cathedral reconstruction shown in Figure 11 should also overlay one of the GPR depth layers to understand the success of the current application.

If any, are all the source data and materials underlying the results available?

Yes

Is the work original in terms of material and argument?

Yes

Is the work clearly and cogently presented?

Yes

Is the argument persuasive and supported by evidence?

Yes

Does the research article contribute to the cultural, historical, social understanding of the field?

Yes

Does it sufficiently engage with relevant methodologies and secondary literature on the topic?

Yes

Reviewer Expertise:

Exploration and computational geophysics

I confirm that I have read this submission and believe that I have an appropriate level of expertise to confirm that it is of an acceptable scientific standard, however I have significant reservations, as outlined above.

# Reviewer report: Reviewer response for version 1

**DOI:** 10.21956/openreseurope.17909.r38785

**Published:** 2024-05-08

**Authors:** Vicente Bayarri

**Affiliations:** 1 Universidad Europea del Atlantico, Santander, Cantabria, Spain

The article presents a study on the application of ground-penetrating radar (GPR) and soil analysis techniques in archaeological prospection, specifically focusing on the identification and mapping of buried features at the site of the destroyed Epiphany Cathedral of Kyiv Brotherhood Monastery. The study aims to provide insights into the archaeological potential of the site and inform conservation and management plans for cultural heritage preservation.

 Major Points:

- Relevance and Alignment with Journal Scope: The article aligns well with the aims and scope of the journal, which focuses on advancing geophysical techniques and soil analytical methodologies in archaeological research. The study contributes valuable insights into the application of GPR and soil analysis in identifying archaeological features, making it highly relevant to the journal's audience.
- Methodological Rigor: The study shows a robust methodology for conducting GPR surveys and interpreting the data to identify buried archaeological features. The authors effectively describe the data collection process, including equipment used, survey parameters, and data processing techniques. This methodological rigor enhances the credibility of the study's findings.
- Clarity and Presentation: The article is generally well-written and organized, with clear sections and figures that aid understanding. The authors effectively communicate their research goals, methods, results, and conclusions, helping with understanding for readers.
- Conclusions and Implications: The conclusions drawn by the authors are well-supported by the results and have significant implications for archaeological research and cultural heritage management. The study highlights the importance of integrating geophysical techniques and soil analysis in archaeological prospection and underscores the potential of non-invasive methods in uncovering hidden archaeological features.

Minor Points:

- Citation of Current Literature: While the article cites relevant literature, there may be opportunities to incorporate more recent publications to summarize current research trends in the field. Updating the literature review section would enhance the article's scholarly impact and relevance.
- Detailed Methodological Information: While the authors explain the GPR survey methods, more information on data processing and analysis techniques could improve the replicability of the study. Providing step-by-step procedures or referencing relevant software/tools used in data analysis would help readers seeking to replicate the study.
- Enhancement of Clarity: Some sections of the article could benefit from further clarification or elaboration to improve understanding, particularly for readers less familiar with geophysical techniques or archaeological terminology. Adding explanatory notes or definitions of key terms would enhance readability and accessibility for a diverse audience.

If any, are all the source data and materials underlying the results available?

Yes

Is the work original in terms of material and argument?

Yes

Is the work clearly and cogently presented?

Yes

Is the argument persuasive and supported by evidence?

Yes

Does the research article contribute to the cultural, historical, social understanding of the field?

Yes

Does it sufficiently engage with relevant methodologies and secondary literature on the topic?

Partly

Reviewer Expertise:

GPR, data integration, data processing, remote sensing, geomatics

I confirm that I have read this submission and believe that I have an appropriate level of expertise to confirm that it is of an acceptable scientific standard, however I have significant reservations, as outlined above.

# Reviewer report: Reviewer response for version 1

**DOI:** 10.21956/openreseurope.17909.r36442

**Published:** 2024-01-02

**Authors:** Nikos Papadopoulos

**Affiliations:** 1 Foundation for Research and Technology - Hellas, Nikolaou Plastira, Greece

The paper demonstrates a case study of urban geophysical prospection in an effort to reveal the foundations of the destroyed Epiphany Cathedral in Kyiv with GPR. The objectives of the work are presented clearly. The historical background is outlined adequately justifying the need of a geophysical prospection survey. The choice of GPR comprises the optimum method for a fast and efficient mapping. The text is relatively clearly written, but further proof editing seems necessary for further refinement (eg from long sentences that convey the meeting). 

 The presentation of radargrams and/or time slices from both the 300 and 700 MHz would be beneficial. Even if the results from the 700MHz are not satisfactory, the potential discussion on this aspect definitely interests the archaeological geophysical community. The unsuccessful experiments can also help to further improve and expand our knowledge.

 The overlay of the GPR survey area and depth slices on the map of Figure 7 would be more appropriate to correlate the reflections with the actual modern buildings. It is not clear why the authors chose to change the background map to overlay their results. The inclusion of more depth slices would be an asset to assess the different reflections in various depths.

 A classification of the reflections related to utilities/modern infrastructures and potential archaeological structures would be beneficial. In this sense it is not quite clear how the diagrammatic interpretation in figure 11 resulted.

 For the conclusions/discussion it could be mentioned how your work progressed the field of urban geophysical prospection in Ukraine, thus placing your effort in a wider context, signifying the impact on the field of cultural resources management.

If any, are all the source data and materials underlying the results available?

Partly

Is the work original in terms of material and argument?

Yes

Is the work clearly and cogently presented?

Partly

Is the argument persuasive and supported by evidence?

Partly

Does the research article contribute to the cultural, historical, social understanding of the field?

Yes

Does it sufficiently engage with relevant methodologies and secondary literature on the topic?

Yes

Reviewer Expertise:

Applied geophysics, archaeological geophysics, geoinformatics in cultural and natural resources managmentn

I confirm that I have read this submission and believe that I have an appropriate level of expertise to confirm that it is of an acceptable scientific standard, however I have significant reservations, as outlined above.
